# Decidua Parietalis Mesenchymal Stem/Stromal Cells and Their Secretome Diminish the Oncogenic Properties of MDA231 Cells In Vitro

**DOI:** 10.3390/cells10123493

**Published:** 2021-12-10

**Authors:** Yasser Basmaeil, Eman Bahattab, Abdullah Al Subayyil, Haya Bin Kulayb, Maha Alrodayyan, Mohammad Abumaree, Tanvir Khatlani

**Affiliations:** 1 Stem Cells and Regenerative Medicine, Cell Therapy and Cancer Research (CTCR), King Abdullah International Medical Research Center (KAIMRC), King Saud Bin Abdulaziz University for Health Sciences (KSAU), King Abdulaziz Medical City, Ministry of National Guard Health Affairs (MNGHA), P.O. Box 22490, Riyadh 11426, Saudi Arabia; basmaeily@ngha.med.sa (Y.B.); alsubayyilab@ngha.med.sa (A.A.S.); hayabinkulayb@gmail.com (H.B.K.); AlrodayyanMa@ngha.med.sa (M.A.); 2 National Center for Stem Cell Technology, Life Sciences and Environment Research Institute, King Abdulaziz City for Science and Technology (KACST), P.O. Box 6086, Riyadh 11442, Saudi Arabia; em.bahattab@gmail.com

**Keywords:** placenta, decidua parietalis MSCs, MDA231, conditioned medium, adhesion, proliferation, migration, invasion, angiogenesis

## Abstract

Mesenchymal stem cells (MSCs) have been shown to suppress tumor growth, inhibit angiogenesis, regulate cellular signaling, and induce apoptosis in cancer cells. We have earlier reported that placenta-derived decidua parietalis mesenchymal stem/stromal cells (DPMSCs) not only retained their functional characteristics in the cancer microenvironment but also exhibited increased expression of anti-apoptotic genes, demonstrating their anti-tumor properties in the tumor setting. In this study, we have further evaluated the effects of DPMSCs on the functional outcome of human breast cancer cell line MDA231. MDA231 cells were exposed to DPMSCs, and their biological functions, including adhesion, proliferation, migration, and invasion, were evaluated. In addition, genomic and proteomic modifications of the MDA231 cell line, in response to the DPMSCs, were also evaluated. MDA231 cells exhibited a significant reduction in proliferation, migration, and invasion potential after their treatment with DPMSCs. Furthermore, DPMSC treatment diminished the angiogenic potential of MDA231 cells. DPMSC treatment modulated the expression of various pro-apoptotic as well as oncogenes in MDA231 cells. The properties of DPMSCs to inhibit the invasive characteristics of MDA231 cells demonstrate that they may be a useful candidate in a stem-cell-based therapy against cancer.

## 1. Introduction

In spite of immense scientific, technological, therapeutic, and medical advances, cancer remains the leading cause of deaths worldwide. An estimated 19.3 million new cancer cases and about 10.0 million cancer deaths worldwide were reported in 2020 alone. This number is expected to rise to about 30 million new patients with almost 17 million deaths worldwide by the year 2040 [1]. The traditional treatment options for cancer, such as chemotherapy in combination with radiotherapy and surgery, remain ineffective for certain tumors, in spite of being the first line of treatment [2,3]. For the past decade, the standard treatments and diagnostics have evolved tremendously, yet the overall success remains abysmal. Tumor specificity and heterogeneity remain the main reasons for the efficient outcome of the traditional therapies [3]. Keeping that scenario in mind, many non-conventional therapies have been proposed and tested against various molecular targets in cancer [4]. They include cancer vaccines, monoclonal antibodies, and cell-based therapies, such as chimeric antigen receptor (CAR) T-cell therapy [5]. Because of their enhanced tumor targeting and minimal off-target effects stem cell therapy is gaining ground and is becoming a popular choice for treatment against cancer [6,7].

Stem cells isolated from adult tissues, the mesenchymal stem/stromal cells (MSCs), have enormous potential to be used in stem-cell-based therapies. MSCs are undifferentiated cells isolated from different tissues and organs, including adipose tissue, bone marrow, umbilical cord, dental pulp, placenta, etc. [8]. They grow on plastic and have self-renewal capabilities. They differentiate into other cell lineages such as adipocytes, chondrocytes, and astrocytes [9,10]. Based on their multifaceted characteristics, including the modulation of immune responses and their unique proliferative and migratory potential, they are considered useful for regenerative medicine and as therapeutic agents against various diseases, including cancer. Many studies have shown that MSCs act in an anti-tumorigenic manner to suppress disease progression. In vivo and in vitro studies have shown that MSCs inhibit tumor growth and metastasis through the modulation of immune responses, inhibition of angiogenesis, regulation of cellular signaling, and/or by inducing apoptosis [11]. 

Various in vitro as well as animal studies have confirmed the anti-tumor effects of MSCs. Multiple studies have demonstrated that MSC therapy decreases the growth of various cancers, including that of the lung, breast, melanoma and glioma, etc. [12,13,14,15]. Subcutaneous MSC injections in melanoma mouse models demonstrated anti-angiogenic properties and initiated apoptosis, resulting in the shrinkage of tumors [16]. In vitro studies using umbilical-cord-derived stem cells showed apoptosis of gliobastoma multiforme (GBM) cell lines mediated through tumor necrosis factor (TNF)-related apoptosis-inducing ligand (TRAIL) in co-culture experiments [17,18]. Additionally, preconditioned MSCs have been shown to express various soluble factors with pro-apoptotic and anti-proliferative properties, making them favorable for targeted therapies against cancer [19]. 

We have earlier reported the isolation and characterization of three types of MSCs from human placentae (pMSCs), which include decidua basalis mesenchymal stem cells (DBMSCs) [20], decidua parietalis mesenchymal stem cells (DPMSCs) [21], and chorionic villus mesenchymal stem cells (CVMSCs) [22]. Their immunomodulatory properties were also studied [23]. More recently, we have reported that DPMSCs retained their functional characteristics (i.e., proliferation, adhesion, migration, and invasion) under sustained exposure to a medium mimicking the cancer microenvironment [24]. In spite of reduced proliferation and adhesion, DPMSCs exhibited enhanced invasive and migratory properties. In addition, continued exposure of DPMSCs to the cancer microenvironment resulted in the increased expression of genes with anti-cancer properties, demonstrating that they not only function normally in the toxic cancer environment but also deliver much-anticipated anti-tumor properties in the tumor setting [24].

Based on these results, and in order to utilize DPMSCs for anti-tumor therapies, it is pertinent to investigate their consequential effects on cancer. Towards that direction, in this study we have evaluated the effects of the cellular component as well as the secretome of DPMSCs on the functional outcomes of human breast cancer cell line MDA-MB-231 (MDA231), and deciphered the basic mechanism behind such changes. In order to observe the specific effects of DPMSCs specifically on human breast cancer cells MDA231 and not on the normal tissues, we used normal human mammary epithelial cells (HMECs) as a control to rule out the negative impact of DPMSCs and its CM on the normal tissues. We performed spatial and temporal treatment of DPMSCs on the MDA231 cell line and evaluated their functional and phenotypic properties, including proliferation, adhesion, migration, and invasion potential. We performed mRNA analysis of some selected genes that play important roles in these functional outcomes. Finally, we analyzed the effect of DPMSCs on MDA231 cells in apoptotic and cell cycle pathways. 

## 2. Materials and Methods

### 2.1. Ethical Approval and Tissue Collection 

The study was approved by the Institutional Review Board (IRB) of King Abdullah International Medical Research Centre (KAIMRC), under proposal number RC20/346/R. Placentae and umbilical cord tissues were collected from human subjects with uncomplicated and healthy pregnancies within 2 h of normal vaginal delivery and with 38–40 weeks of gestation. Informed consent was received from the donors before collection of the placenta. Fetal age and viability were regularly monitored during the gestational period by ultrasound examinations. Research guidelines set by the IRB were strictly followed during the clinical and experimental procedures. 

### 2.2. Cell Lines, Antibodies, and Reagents 

The MDA-MB-231 (MDA231) breast cancer cell line (cat#HTB-26) was purchased from American Type Culture Collection (ATCC, Manassas, VA, USA). Human mammary epithelial cells (HMECs) (cat#A10565) were procured from Thermo Fisher Scientific (Waltham, MA, USA). Fluorescent-labeled antibodies for flow cytometry experiments, including GAPDH (cat#AF5718); ICAM (cat#BBA20); PECAM (cat#FAB3567P); integrin α5 (cat#FAB1864P); and EpCAM (cat#FAB9601P) were purchased from R&D Systems (Minneapolis, MN, USA). Antibodies for immunoblotting—P53 (cat#2527), pRb (S807) (cat#8516), Chk1 (cat#2348), cleaved caspase-3 (cat#9664), and β-actin (cat#3700 and cat#8457)—were purchased from Cell Signaling Technologies (Danvers, MA, USA). Anti-rabbit and anti-mouse secondary antibodies (cat#21537 and 21538M) were purchased from Sigma-Aldrich (St. Louis, MO, USA). Protease inhibitor cocktail (cat#P8340) was purchased from Millipore Sigma (Burlington, MA, USA).

### 2.3. Cell Culture and Conditioned Media (CM) Collection

MDA231 cells were cultured in Dulbecco’s Modified Eagle’s Medium/Nutrient Mixture F-12 (DMEM-F12) supplemented with 2% penicillin-streptomycin (PEST) (Thermo Fisher Scientific, Waltham, MA, USA), 10% FBS, and 1% GlutaMax (Gibco^®^, Life Technologies, Waltham, MA, USA) in T-75 tissue culture flasks. HMECs were cultured in MEGM Mammary Epithelial Cell Growth Medium BulletKit (cat#CC-3150) (Lonza, Basel, Switzerland) containing 10% FBS. All cell lines were incubated at 37 °C in humidified air with 5% CO_2_ and 95% air. The cell medium was changed every 2–3 days, and the cells were passaged with TrypLE Express, Life Technologies, Carlsbad, CA, USA, at 75–90% confluence.

DPMSCs were isolated from the decidua parietalis region of the placenta (lining the main cavity of the pregnant uterus) using the enzyme digestion method, as described earlier [21]. In brief, 10 g of the choriodecidua tissue was manually separated from the amnion, rinsed with phosphate-buffered saline (PBS; pH of 7.4) and minced thoroughly into a tissue paste in PBS. The tissue pellet was collected after centrifugation and incubated with 0.05% trypsin-EDTA (cat#25300054) Life Technologies, Carlsbad, CA, USA and 270 unit/mL DNase I (cat# 18047019) Life Technologies, Carlsbad, CA, USA for 15 min at 37 °C. The digested tissue pellet was washed in Dulbecco’s Modified Eagle’s Medium/Nutrient Mixture F-12 (DMEM-F12) containing 10% fetal bovine serum (FBS) for 10 min. After centrifugation the supernatant was discarded and the tissue was strained through a 100 µm straining filter, then washed with PBS and centrifuged. After discarding the supernatant, the pellet was incubated in RBC lysing buffer (cat#sc-3621, Santa Cruz, CA, USA) for 45 min at room temperature. The cells were pelleted and suspended in a DMEM-F12 medium containing 10% MSC Certified FBS (MSC-FBS cat# 12662029, Thermo Fisher Scientific, Waltham, MA, USA), and placed in a cell culture incubator (37 °C, 5% CO_2_, and 95% air). Cells were harvested and characterized before use. Cells at passage 3 were used in subsequent experiments.

Human umbilical vein endothelial cells (HUVECs) were isolated from umbilical cord veins using already-published methods [25]. Briefly, the umbilical veins were washed thoroughly with PBS, before digesting them with collagenase type II (cat# 17101-015, ThermoFischer Scientific, Riyadh, Saudi Arabia) in PBS solution. The tissue was incubated for 25 min at 37 °C in a humidified incubator with 5% CO_2_ and 95% air. The liberated HUVECs were collected and resuspended in complete Endothelial Cell Growth Medium (cat# PCS-100-041™, ATCC, Manassas, VA, USA) and cultured at 37 °C in a cell culture incubator. The cells were characterized by flow cytometry before being used in the subsequent studies.

The conditioned medium (CM) was harvested from the culture of DPMSCs using our previously published method [24]. Briefly, 1 × 10^5^ DPMSCs were cultured in a DMEM-F12 culture medium with 10% MSC-FBS, 100 μg/mL L-glutamate, and antibiotics (100 U/L penicillin and 100 μg/mL streptomycin) until cells reached 75% confluency. Cells were washed with PBS before feeding them with fresh medium and further incubated for 72 h (h). The conditioned medium (CM-DPMSCs) was collected, centrifuged to remove the dead cells, and stored at −80 °C for future use.

### 2.4. Treatment of MDA231 Cells and HMECs with DPMSCs 

For direct cell–cell contact (IC) experiments, the culture system consisted of DPMSCs seeded in the reverse side of a 0.4 µm pore size membrane and MDA231 cells/HMECs seeded on the upper chamber (at a ratio of 1:1, 1:2, and 1:4 between DPMSCs to MDA/HMECs) of the membrane in a complete medium. Cells were incubated for 24 h at 37 °C in a cell culture incubator. For the soluble factor (SF) setting, the co-culture system consisted of MDA231 cells/HMECs seeded in the bottom of a 6-well plate and DPMSCs seeded in 0.4 μm pore size transwell membrane (cat# 9300402, cellQART, Northeim, Germany) for 24 h at a DPMSCs:MDA231 cells/HMECs ratio of 1:1, 1:2, and 1:4. For the treatment of MDA231 cells/HMECs with CM-DPMSCs, the CM was initially diluted in a complete medium to a working solution of 20%, 30%, and 40%. The diluted CM was then added to MDA231 cells/HMECs, and the cells were incubated for 72 h at 37 °C, before performing the functional and other assays.

### 2.5. Cellular Proliferation by MTS Assay 

The proliferation of MDA231 cells and HMECs treated with DPMSCs and untreated controls was measured with an MTS kit (CellTiter 96 Aqueous Non-Radioactive Cell Proliferation Assay, cat#G5421, Promega, Germany). MDA231 cells/HMECs were cultured with DPMSCs at different ratios and/or different concentrations of CM-DPMSCs at DPMSCs:MDA231 cells/HMECs (1:1, 1:2, 1:4, and 2:1) and CM-DPMSCs at 20%, 30%, and 40%. All cultures were performed in a culture medium as described above. MDA231 cell/HMEC proliferation was then assessed after 72 h of treatment using an MTS kit as instructed by the manufacturer. Briefly, the cells were incubated in an MTS solution for 4 h at 37 °C and the absorbance was recorded at 490 nm using an ELISA plate reader (Spectra MR, Dynex Technologies, Denkendorf, Germany). Results from triplicate samples were presented as mean ± standard errors. DPMSCs used in these experiments were initially treated with 25 μg/mL mitomycin C at 37 °C for 1 h to cease their proliferation, as previously described [24].

### 2.6. xCELLigence: Impedence-Based Assays

Various cellular functions, including the adhesion, proliferation, migration, and invasion, of MDA231 cells treated with or without DPMSCs were examined using an xCELLigence Real-Time Cell Analyzer system (RTCA-DP version; Roche Diagnostics, Mannheim, Germany). This is a widely used real-time cell analysis system that monitors continuous cellular events by recording label-free changes in electrical impedance, which is reported as the cell index [26,27,28].

For proliferation and adhesion, “E-Plate 16” (cat#05469813001, Roche Diagnostics, Basel, Switzerland) was used. Briefly, 100 μL of medium (with or without CM-DPMSCs) was added to each well of the plate and background impedance was recorded, as previously described [29]. The cells of each experimental group (treated with DPMSCs and untreated control) were seeded in four wells, and the plate was incubated at room temperature for 30 min (in order to reach an equilibrium) before placing it in the xCELLigence system at 37 °C in a cell culture incubator. The cell index was monitored for 72 h. Cellular adhesion was measured after 2 h, and the rate of cell proliferation was calculated after 72 h. Data were analyzed using RTCA xCELLigence, (Roche, Basel, Switzerland) software (version 1.2.1), and final data for proliferation were demonstrated after normalizing them with the adhesion data. Data for both adhesion and proliferation are expressed as mean normalized cell index with ± standard errors. 

Specially designed 16-well plates, “CIM-16” (cat#05665825001, Roche Diagnostics, Basel, Switzerland), were used to record the migration potential of DPMSC-treated and untreated MDA231 cells. The CIM-16 migration plates have upper and lower chambers separated by a porous polyethylene terephthalate (PET) membrane (pore size of 8 μm) in conjunction with microelectrodes [29]. To the wells of the upper chamber 50 μL of pre-warmed serum-free media was added, and the plates were placed in the RTCA device and incubated at 37 °C in a cell culture incubator for 1 h to obtain equilibrium. MDA231 cells (treated or untreated controls) were seeded at a density of 20 × 10^3^ in the upper chamber of the plate in 100 μL of medium. The plates were incubated at room temperature for 30 min to allow the cells to adhere to the membrane. A complete medium supplemented with 10% FBS was added to the lower chamber as a chemo-attractant for the migrating cells. The impedance value of each well was captured by the xCELLigence system after every 15 min for 24 h, as described above. 

For the monitoring of cellular invasion, HUVECs at a density of 2 × 10^4^ cells were added to a 16-well culture E-Plate to create a monolayer of cells. DPMSC-treated or untreated control MDA231 cells at a density of 1 × 10^4^ cells were added to the HUVEC monolayer, as described earlier [30]. After 48 h, the cell invasion index (mean ± standard errors) was measured by calculating the normalized cell index at pausing time (15–20 h) of HUVEC growth. All experiments were performed with four sets of MDA231 cells treated independently with DPMSCs isolated from four different placentae.

### 2.7. Cell Migration, Invasion, and Angiogenesis Assays

The effect of DPMSCs on the migratory, invasiveness, and angiogenesis potential of MDA231 cells was further evaluated by the transwell and tube formation assays. For the migration assay, the cells were made to pass through 8 mm pore polycarbonate transwell inserts, and for invasion assay, the inserts were coated with Matrigel (cat#356235, BD Biosciences, San Jose, CA, USA). DPMSC-treated and untreated MDA231 cells were seeded with serum-free DMEM-F12 medium at a concentration of 2.5 × 10^3^ cells/mL in the upper chamber of the inserts. A complete medium with 20% FBS was used as a chemo-attractant added to the lower chamber of the plate. After incubation for 24 h in a humidified cell incubator, the migrated/invaded cells were washed with PBS and fixed with 4% paraformaldehyde for 15 min at room temperature. The cells were stained with 0.1% crystal violet and visualized as well as photographed under a light microscope (250× magnification). The rate of migration and invasion was determined after counting the migrated/invaded cells.

For the tube formation assay, 100 μL of Matrigel (cat#CLS356230, Merck, St. Louis, MO, USA) was plated into individual wells of a 96-well tissue culture plate and allowed to polymerize overnight at 37 °C in a cell culture incubator. Five experimental groups were used in the tube formation experiments. They included the DPMSCs as a control; the HUVECs as a positive control for tube formation; untreated MDA231 cells; MDA231 cells cultured in 40% CM-DPMSCs; and MDA231 cells co-cultured with DPMSCs at a ratio of 1:1. The cells were seeded at a density of 3 × 10^4^ cells per well in a complete medium on the polymerized Matrigel and incubated for 14 h for tube formation. The tubes were visualized under an inverted Nikon ECLIPSE Ti U microscope (Nikon, Japan). Photomicrographs were recorded using a Nikon DS-Qi1 camera (Nikon, Japan), and nodes of the capillary network were counted. The results were presented as mean ± standard deviation. Experiments were conducted in triplicate and repeated at least three times. 

### 2.8. RNA Expression Profiling by Real-Time PCR Analysis (RT-qPCR)

We used a Human Breast Cancer RT^2^ Profiler Kit (cat# PAHS-131Z, Qiagen, Hilden, Germany) to perform RNA expression profiling of MDA231 cells after treatment with DPMSCs or without treatment in a real-time polymerase chain reaction (RT-PCR) assay.

Total RNA was isolated from treated and untreated MDA231 cells using an RNEasy Mini Kit (cat#74104, Qiagen, MD, USA). It was transcribed into single-stranded cDNA using the FastLane cDNA Analysis Kit (cat#215011, Qiagen, MD, USA). An RT-PCR reaction was performed to detect the expression of 84 genes related to human breast cancer using a CFX96 real-time PCR detection system (Bio-Rad, Hercules, CA, USA). Data were analyzed by calculating ΔΔ^−2^ values and expressed as fold change expression, as compared to the relative expression of housekeeping genes (GAPDH or β-actin) used as an internal control. Experiments were performed in triplicate and repeated three times using DPMSCs isolated from three different placentae.

### 2.9. Flow Cytometry

MDA231 cells treated with DPMSCs or untreated controls were harvested, and 1 × 10^5^ cells were stained using fluorescent-conjugated monoclonal antibodies against the specific adhesion molecule as described above in the “antibodies and reagents” section, and as described before [30]. Briefly, the cells were incubated with respective antibodies for 30 min and washed with cold PBS at 8 °C. For the intracellular expression of the proteins, the cells were fixed with 4% paraformaldehyde in sterile PBS for 10 min at room temperature (RT), and permeabilized for 10 min at RT in PBS containing 0.1% saponin. Intracellular and cell-surface protein expression was assayed by a BD FACS CANTO II (Becton Dickinson, Bergen County, NJ, USA) flow cytometer. For significant detection of expression, 10^4^ to 10^7^ events were collected, stained, gated, and included in the graphical analysis of the data. The cells stained with PE-conjugated Mouse IgG1 kappa Isotype Control (P3.6.2.8.1), cat # 12-4714-82, and/or FITC-conjugated Mouse IgG1 kappa Isotype Control (P3.6.2.8.1), cat # 11-4714-81 (Thermo Fisher Scientific, Waltham, MA, USA) were used as a negative control. 

### 2.10. Immunoblotting

DPMSC-treated MDA231 cells and untreated control cells were washed with PBS and lysed in 100 μL of cell lysis buffer (Cat#9803, Cell Signaling Technologies, MA, USA) containing protease and phosphatase inhibitors (wherever needed). Lysed cell suspension was centrifuged at 15,000 rpm for 5 min at 4 °C; the supernatant was collected and stored at −80 °C, for further use. Protein concentration was estimated by the Bradford assay method before electrophoresis. Thirty micrograms of lysate in an equal amount (*v*/*v*) of 2× Laemmli Sample Buffer (Cat#1610737, Bio-Rad, CA, USA) was denatured at 99 °C for 10 min before being loading onto a 10% SDS-PAGE gel. Resolved proteins were transferred onto a nitrocellulose membrane using a Mini Trans-Blot System (Bio-Rad, Hercules, CA, USA) at 100 V for 120 minutes. Non-specific antigens were blocked by Tris-buffered saline with 0.1% (*v*/*v*) Tween 20 (TBS-T) and 5% non-fat dry milk for 30 min at RT. Primary antibodies at a 1:1000 dilution in a TBS-T buffer containing 5% skimmed milk or BSA were added to the membranes and incubated overnight at 4 °C with continuous shaking. After being washed three times with TBS-T, the membranes were incubated with horseradish peroxidase (HRP)-conjugated anti-goat or anti-rabbit secondary antibodies (R&D Systems, Minneapolis, MN, USA) at a 1:3000 dilution for 2 h at room temperature. The blots were washed three times with TBS-T, and the bands were visualized using SuperSignal™ West Pico (Cat#34577) or West Femto Chemiluminescent Substrate (Cat#34096, Thermo Fisher Scientific, Waltham, MA) in a ChemiDoc^®^ visualization system (Bio-Rad, Hercules, CA, USA). Image-analyzing software, Image Lab (Bio-Rad, Hercules, CA, USA), was used to measure the density of the bands that were normalized with the density of bands obtained for β-actin as a loading control. The experiments were repeated three times using cells treated with DPMSCs isolated from three different placentae. 

DPMSC-treated MDA231 cells and untreated control cells were washed with PBS and lysed in 100 μL of cell lysis buffer (Cat#9803, Cell Signaling Technologies, MA, USA) containing protease and phosphatase inhibitors (wherever needed). Lysed cell suspension was centrifuged at 15,000 rpm for 5 min at 4 °C; the supernatant was collected and stored at −80 °C, for further use. Protein concentration was estimated by the Bradford assay method before electrophoresis. Thirty micrograms of lysate in an equal amount (*v*/*v*) of 2x Laemmli Sample Buffer (Cat#1610737, Bio-Rad, CA, USA) was denatured at 99 °C for 10 min before being loading onto a 10% SDS-PAGE gel. Resolved proteins were transferred onto a nitrocellulose membrane using a Mini Trans-Blot System (Bio-Rad, Hercules, CA, USA) at 100 V for 120 minutes. Non-specific antigens were blocked by Tris-buffered saline with 0.1% (*v*/*v*) Tween 20 (TBS-T) and 5% non-fat dry milk for 30 min at RT. Primary antibodies at a 1:1000 dilution in a TBS-T buffer containing 5% skimmed milk or BSA were added to the membranes and incubated overnight at 4 °C with continuous shaking. After being washed three times with TBS-T, the membranes were incubated with horseradish peroxidase (HRP)-conjugated anti-goat or anti-rabbit secondary antibodies (R&D Systems, Minneapolis, MN, USA) at a 1:3000 dilution for 2 h at room temperature. The blots were washed three times with TBS-T, and the bands were visualized using SuperSignal™ West Pico (Cat#34577) or West Femto Chemiluminescent Substrate (Cat#34096, Thermo Fisher Scientific, Waltham, MA) in a ChemiDoc^®^ visualization system (Bio-Rad, Hercules, CA, USA). Image-analyzing software, Image Lab (Bio-Rad, Hercules, CA, USA), was used to measure the density of the bands that were normalized with the density of bands obtained for β-actin as a loading control. The experiments were repeated three times using cells treated with DPMSCs isolated from three different placentae. 

### 2.11. Data Analyses

The statistical analyses were performed using GraphPad Prism ver 8.0 (GraphPad Software, La Jolla, CA, USA) and SPSS 22.0 (IBM, SPSS, Chicago, IL, USA). Each experiment was repeated at least three times to avoid experimental bias. Single-factor data between two groups were compared by one-way analysis of variance (ANOVA), while data of double factors in multiple groups were compared by two-way ANOVA. An unpaired t-test was applied for data comparison between two groups. Data are shown in bar graphs as means of values with ± standard error (SE) obtained from three independent experiments. A *p*-value of ≤0.05 was considered to be statistically significant. 

## 3. Results

### 3.1. Temporal and Dose–Response Standardization

We evaluated the suitable concentration of CM-DPMSCs and the appropriate number of DPMSCs, which had a measurable effect on the basic functionality of the MDA231 cells. Initially, we have selected three ratios—1:1; 1:2, and 1:4—for DPMSCs to MDA231 cells/HMECs. For conditioned media treatment, the CM-DPMSCs doses at 20%, 30%, and 40% we selected. For cellular contact, the MDA231 cells or HMECs were treated in both intracellular (IC) and secretory factor (SF) settings. The cells were incubated for 24 h, 48 h, and 72 h to calculate the appropriate time where the maximum effect would be observed. An MTS assay was performed on the treated cells.

**Figure 1:**
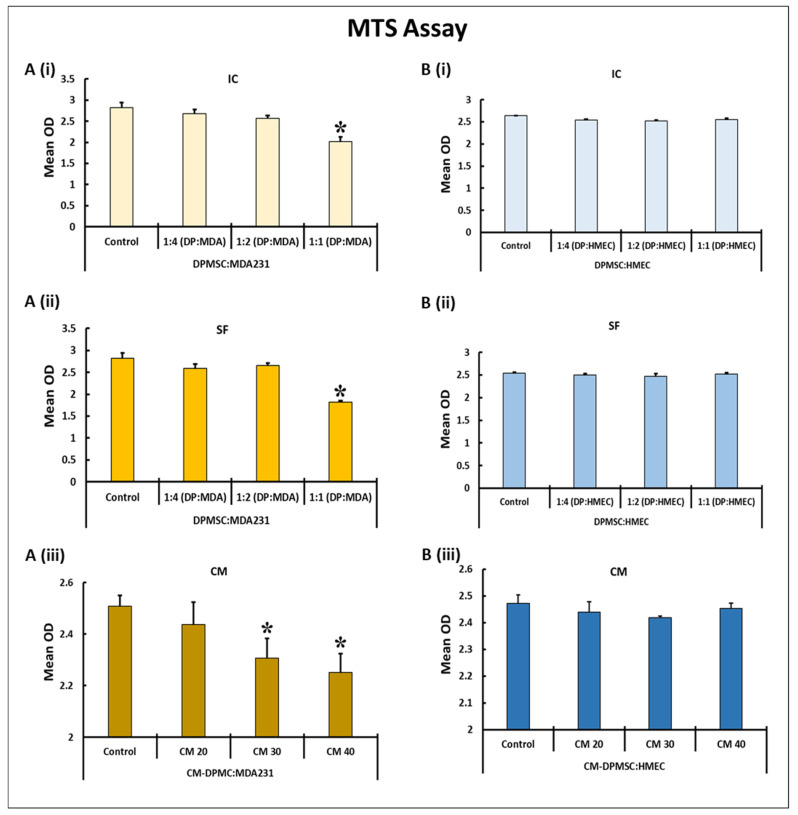
Dose curve of DPMSCs and CM for treatment of MDA231 cells and HMECs: The effect on MDA231 cells (**A**) and HMECs (**B**) in response to different ratios of DPMSCs and different concentrations of CM-DPMSCs at 72 h, post-treatment, using an MTS assay system. After treatment, the proliferation of MDA231 cells did not change in response to the ratios of 1:2 and 1:4 pertaining to DPMSCs to MDA231 cells, as compared to untreated controls. At a 1:1 ratio MDA231 cell proliferation reduced significantly in the IC setting (**A**(**i**)) as well as in the SF setting (**A**(**ii**)) as compared to untreated control groups. MDA231 cells demonstrated a significant reduction in growth rate when treated with CM at 30% as well as at 40% concentration as compared to 20% CM treatment and the untreated control (**A**(**iii**)). In comparison, DPMSCs did not have any effect on HMEC cell proliferation in the IC (**B**(**i**)) or SF (**B**(**ii**)) settings as well as at various concentrations of CM of DPMSCs tested (**B**(**iii**)). IC: intracellular contact; SF: soluble factors; and CM: conditioned media. Mean OD is the mean of absorbance obtained from three individual experiments. Bars represent standard errors. * *p* < 0.05.

Both MDA231 cells and HMECs incubated for 24 h and 48 h either with CM-DPMSCs or the cellular component of DPMSCs did not show any change in their phenotypes (data not shown). Rather, as shown in Figure 1A(i),B(i), the co-cultured MDA231 cells in the IC setting did not show any change in proliferation at 1:2 and 1:4 concentrations for both HMECs as well as MDA231 cells. However, at a 1:1 concentration, the MDA231 cells showed a significant reduction (*p* < 0.05) in proliferation as compared to the untreated control. As expected, this effect was not observed for HMECs treated under similar conditions with DPMSCs (Figure 1A(i)). A similar phenotype was observed when the cells were co-cultured in the SF setting. As shown in Figure 1A(ii),B(ii), the cellular ratios at 1:2 and 1:4 for DPMSCs to MDA231 cells/HMECs did not exhibit any change in the growth behavior between the control and various treatment settings. However, as observed in the IC setting, the cellular ratio at 1:1 for DPMSCs to MDA231 cells in the SF setting also showed a significant reduction (*p* < 0.05) in the MDA231 proliferation as compared to the untreated control. As shown in Figure 1A(ii), this phenotype was not observed in DPMSC-treated HMECs. 

In order to determine the appropriate dose of CM-DPMSCs, which has a measurable effect on the functional characteristics on MDA231 cells, three doses of CM-DPMSCs at 1%, 10%, and 20% were initially selected to treat HMEC and MDA231 cells. The cells were subjected to sustained treatment for three different time points at 24 h, 48 h, and 72 h. However, observing no significant changes in any of the CM doses or the time points (data not shown), the doses of CM-DPMSCs were increased to 30% and 40% and the test cells were treated for 72 h continuously, after which an MTS assay was performed. Figure 1A(iii) shows that the CM of DPMSCs did not modulate the proliferation of HMECs at any of the doses tested at any of the selected time points selected. In MDA231 cells, and as shown in Figure 1B(iii), after 72 h of sustained treatment with CM-DPMSCs the cells showed a dose-dependent reduction in overall proliferation, which reached a significant level (*p* < 0.05) at 30% CM-DPMSCs against 20% and the untreated control. This response was more robust for 40% CM-DPMSCs treated MDA231 cells incubated for 72 h, where a strong and significant decrease in proliferation was observed as compared to 20%, 30%, and the untreated control (Figure 1A(iii)). Based on the results obtained above, an exposure time of 72 h was chosen for the treatment of MDA231 cells with 40% CM-DPMSCs, and the ratio was chosen as 1:1 (for both IC and SF), to study their functional outcome.

### 3.2. Impact of DPMSCs (CM and Cells) on the Adhesion and Proliferation of MDA231 Cells

xCELLigence Real-Time Cell Analyzer (RTCA) was used to measure the effect of DPMSCs and their conditioned medium over MDA231 cells. After sustained incubation of MDA231 cells for 72 h with DPMSCs at a ratio of 1:1 at IC and SF settings, as well as at 40% concentration of CM-DPMSCs, the cells were washed with PBS before being harvested and subjected to cell analysis by xCELLigence RTCA. Untreated cells in a complete medium served as the control. The cell viability assay of MDA231 cells treated with the cellular component of DPMSCs at various ratios or with 40% CM-DPMSCs was found at 95%. MDA231 cells treated with higher concentrations of CM-DPMSCs showed decreased viability at >90%. In order to decipher the mechanism of action of the cells and the CM of DPMSCs, an exposure time of 72 h was chosen for the treatment of MDA231 cells with DPMSCs and 40% of CM-DPMSCs to study their functional consequences.

**Figure 2:**
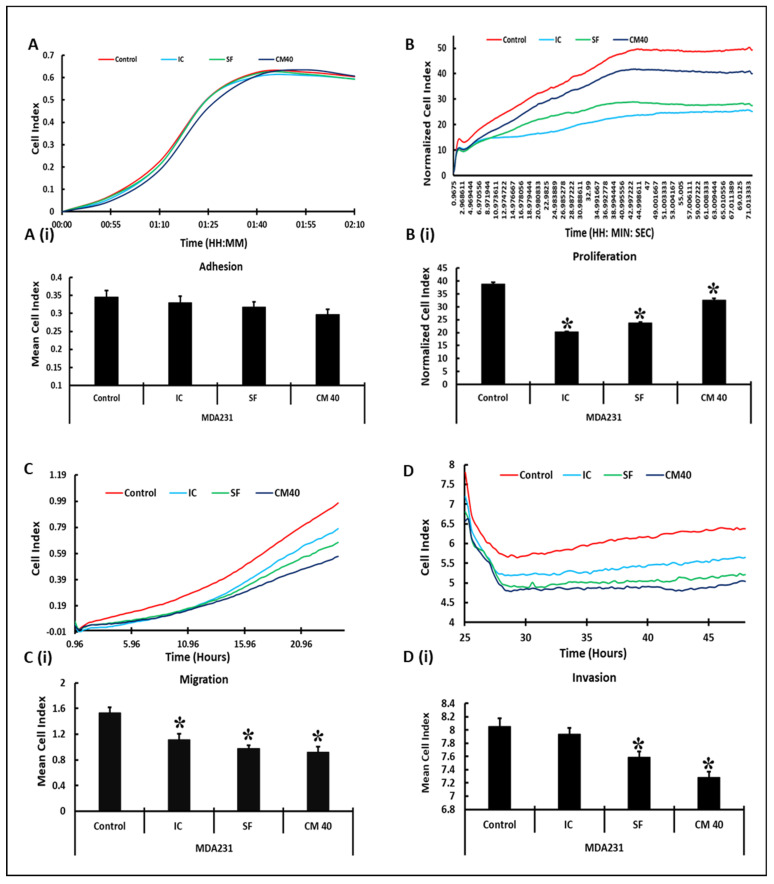
Effect of DPMSCs on MDA231 cell functions: MDA231 cell functions pertaining to cellular adhesion, proliferation, migration, and adhesion, as evaluated by an xCELLigence RTCA system. In response to 1:1 (DPMSCs:MDA231 cells) cellular ratios in the SF setting, and at 40% CM of DPMSCs, MDA231 cell adhesion decreased as compared to the IC setting and the untreated control ((**A**) and (**A**(**i**))). The proliferation of MDA231 cells decreased significantly after treatment with DPMSCs at a 1:1 ratio in IC and SF settings, as well as when treated with 40% CM, as compared to the untreated control ((**B**) and (**B**(**i**))). MDA231 cell migration decreased significantly when treated with the cellular component of DPMSCs in both IC and SF settings, as well as when treated with the CM of DPMSCs, as compared to the untreated control ((**C**) and (**C**(**i**))). The invasion of MDA231 cells did not change significantly when treated with DPMSCs in the IC setting, but in the SF setting as well as after treatment with 40% of CM the invasiveness of MDA231 cells decreased significantly as compared to the untreated control cells ((**D**) and (**D**(**i**))). Each experiment was repeated three times with DPMSCs isolated from five different placentae. Bars represent standard errors. * *p* < 0.05.

As shown in Figure 2A, the adhesion of MDA231 cells did not change significantly when treated with DPMSCs at a ratio of 1:1 in both IC as well as in SF settings in xCELLigence RTCA assays. However, treatment with 40% CM-DPMSCs reduced the adhesion of MDA231 cells significantly (*p* < 0.05) as compared to IC, SF, and untreated control groups. Figure 2A(i) shows the mean cell index recorded 2 h after the start of the experiment, comparing the untreated control MDA231 cells with different treated groups.

MDA231 cells exhibited a significant reduction (*p* < 0.05) in proliferation measured after 72 h of co-culturing with DPMSCs in IC and SF settings compared to the untreated control in an xCELLigence RTCA system (Figure 2B). Although 40% CM-DPMSCs diminished the proliferation of MDA231 cells significantly as compared to the untreated control, the overall reduction in proliferation was not similar to the co-culture settings. As shown in Figure 2B(i), the growth curves obtained in the xCELLigence RTCA corresponded to the normalized cell indices calculated for all experimental groups, where MDA231 cells show a significant reduction in overall proliferation in the IC and SF co-culture as well as with treatment of 40% CM-DPMSCs for 72 h.

### 3.3. DPMSCs Restrain the Invasive Characteristics of MDA231 Cells

As compared to the untreated control, the MDA231 cells co-cultured with DPMSCs in a ratio of 1:1 for DPMSCs to MDA231 cells showed a significant decrease (*p* < 0.05) in migration, as evaluated in an xCELLigence RTCA system (Figure 2C). Similarly, MDA231 cells treated with 40% CM-DPMSCs also showed a significant decrease (*p* < 0.05) in cellular migration as compared to the untreated control, similar to IC and SF settings (Figure 2C). The mean cell index was calculated from the xCELLigence RTCA data and reflected the similar trends of a significant decrease in the cellular migration of MDA231 cells against the treatment groups (Figure 2C(i)).

The cellular invasion of MDA231 cells co-cultured with DPMSCs in IC and SF settings or their treatment with 40% CM-DPMSCs showed a significant reduction (*p* < 0.05) in SF and CM treatment settings as compared to the untreated control (Figure 2D). As compared to the SF co-culture, although the IC setting showed a decreased invasiveness of MDA231 cells as compared to the untreated control, the reduction was not statistically significant (*p* > 0.05). The mean cell index of the invading cells reflected the trend, calculated from the data obtained in the xCELLigence RTCA analysis (Figure 2D(i)).

**Figure 3:**
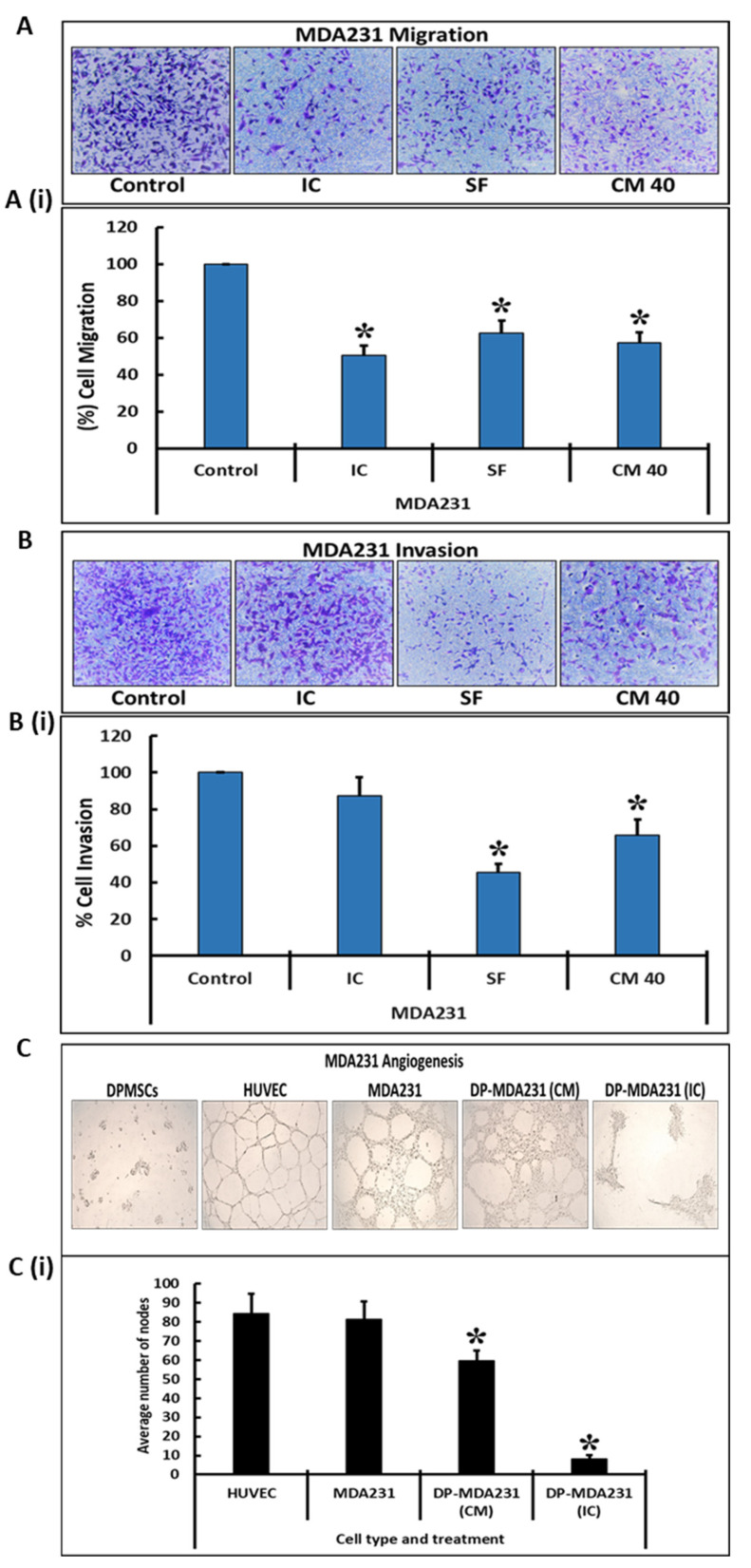
Effect of DPMSCs on MDA231 cell functions: The migration of MDA231 cells after treatment with DPMSCs in IC and SF settings, and after treatment with 40% CM, by a transwell assay. Treated MDA231 cells with DPMSCs in IC and SF settings, as well with 40% CM of DPMSCs, migrated at a significant slower rate through the 8 µM pore of a transwell filter as compared to the untreated control cells. Panel (**A**) shows the photomicrographs of the migrated cells under various treatment conditions. The percentage of migrated cells is presented in a bar graph (**A**(**i**)). The invasion of MDA231 cells treated with DPMSCs in IC and SF settings, as well as with 40% CM of DPMSCs was examined by a Matrigel-coated transwell insert with a pore size of 8 µM, as described in the Material and Methods section. MDA231 cells treated with DPMSCs in the SF setting, as well as the cells treated with 40% CM, showed a significant reduction in cellular invasion as compared to the IC treatment and to the untreated control experiment groups. Panel (**B**) shows the photomicrographs of MDA231 cells that invaded through the Matrigel-coated membrane cells under different treatment conditions. The percentage of invaded cells is presented as a bar graph (**B**(**i**)). DBMSCs did not form the vascular tubes, rather HUVECs (as control) and MDA231 cells formed the tubes on the Matrigel-coated plates. The CM of DPMSCs, as well as with their cellular component, significantly reduced the tube formation capability of MDA231 cells (**C**). The bar diagram represents the average number of tubal nodes calculated from five different fields (**C**(**i**)). Scale bars, 100 μM. Values are represented as means ± SE, * *p* < 0.05.

Similar results were observed in a transwell assay performed to assess the invasive and migratory potential of the MDA231 cells after treatment with cellular and secretory components of the DPMSCs. After co-culturing for 72 h in IC and SF settings, or treatment with 40% CM of DPMSCs, the cells were made to pass through an insert with a pore size of 8 µM for migration assays and Matrigel-coated inserts of the same pore size for invasion assays. As shown in Figure 3A, as compared to the untreated control the co-culture of MDA231 cells with DPMSCs in the IC and SF settings exhibited significantly reduced (*p* < 0.05) the number of cells that migrated through the membrane (Figure 3A(i)). Similarly, the treatment of MDA231 cells with CM-DPMSCs for 72 h and subsequent transwell assay demonstrated a significantly reduced (*p* < 0.05) rate of migration (Figure 3A,A(i)). This is in agreement with the results obtained in the xCELLigence RTCA assays.

The invasion of MDA231 cells treated with the cellular component and CM of DPMSCs was further examined by a transwell assay using the Matrigel-coated membrane inserts. The infiltration of MDA231 cells (which are highly invasive in nature) through the Matrigel-coated membrane defines their invasion potential. The invaded cells were stained with crystal violet and counted. As compared to the untreated control, the invasion of MDA231 cells co-cultured with DPMSCs in the IC setting for 72 h did not change significantly (*p* > 0.05). However, MDA231 cells co-cultured with DPMSCs in the SF setting for 72 h as well as the MDA231 cells treated with 40% CM-DPMSCs for 72 h, exhibited a significant reduction (*p* < 0.05) in invasion across the Matrigel-coated membrane (Figure 3B,B(i)), indicating that DPMSCs alter the invasive potential of MDA231 cells. 

MDA231 cells have the ability to form tubes in the angiogenesis assay, while this feature is absent in the placental mesenchymal stem cells that include the DPMSCs [23]. In order to ascertain the effect of DPMSCs on the tube formation feature of MDA231 cells, we performed an angiogenesis assay. As shown in Figure 3C, the tube formation capability of MDA231 cells was obliterated after treatment with 40% CM-DPMSCs or the cellular component of DPMSCs. Figure 3C(i) shows the reduction in the number of nodes in the treated experimental groups as compared to the untreated controls: MDA231 cells and HUVECs.

### 3.4. Modulation of Functionally Relevant Effectors in MDA231 Cells in Response to DPMSCs

The expression of the important molecules responsible for the modulation of the functional characteristics of MDA231 cells, after treatment with DPMSCs, was studied by RT-PCR analysis and by flow cytometry. An RT^2^ Profiler™ PCR Array Human Breast Cancer kit was used to estimate the expression of proteins involved in oncogenesis and cancer progression. Table 1 depicts the modulation in the expression of numerous molecules in MDA231 cells after treatment with either the cellular component or secretome of DPMSCs. These molecules have either tumor suppressor or oncogenic properties. Various tumor suppressor genes, including BRCA1, BRCA2, FOXA1, GATA3, PTEN, RARB, etc., were upregulated in MDA231 cells after treatment with DPMSCs in both IC and SF settings, as well as when treated with 40% CM of the DPMSCs. In addition, a number of effector molecules, such as MUC1, XBP1, CTSD, ABCB1, etc., were downregulated in MDA231 cells after treatment with DPMSCs in IC and/or SF settings, or their secretome at 40% concentration. 

**Figure 4:**
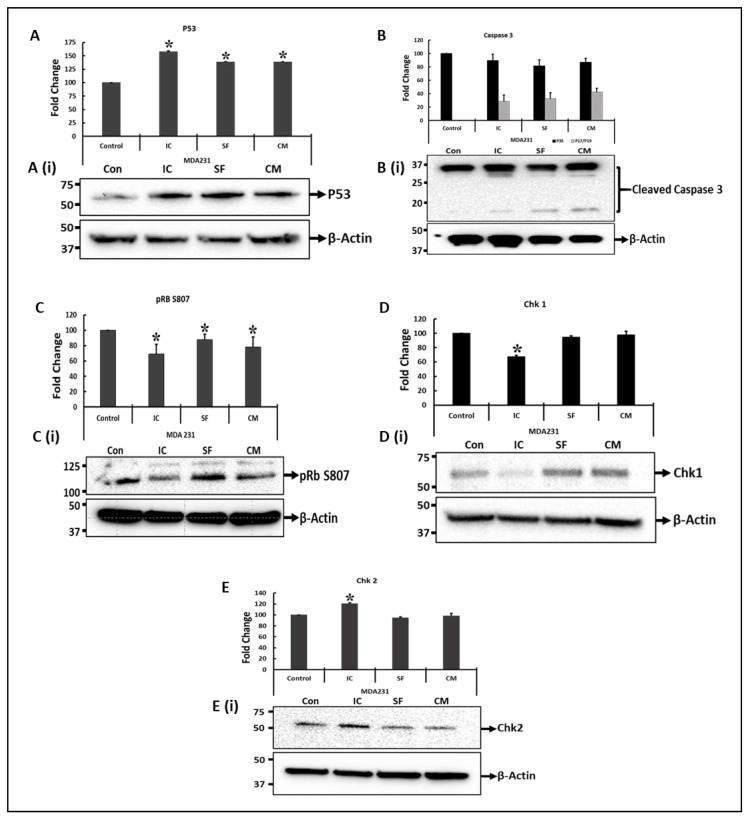
Immunoblotting of cell-cycle-associated proteins: Immunoblotting of selected proteins involved in important cellular functions was performed on MDA231 cells after treatment with DPMSCs. Expression levels of p53 increased significantly in IC and SF settings, as well as after treatment with 40% CM of DPMSCs (**A**). After treatment, caspase-3 cleaved into P17 and P19 components (**B**). The phosphorylation of the Rb protein at S807, as well as the total levels of Chk1 proteins, decreased significantly in IC settings as compared to control, SF and CM (**C**,**D**), whereas Chk2 protein expression levels increased significantly in MDA231 cells after treatment with DPMSCs (**E**). Bars at (**A**(**i**), **B**(**i**), **C**(**i**), **D**(**i**), and **E**(**i**)) represent the density of the bands, measured using Image Lab^®^ software. Values are represented as means ± SE, * *p* < 0.05.

In addition, we analyzed the expression of a few cell cycle proteins in MDA231 cells involved in cell cycle progression and/or apoptosis after their treatment with DPMSCs. Figure 4 shows that the expression of p53 increased significantly (*p* < 0.05) in MDA231 cells treated with DPMSCs in IC and SF settings, as well as with 40% CM of DPMSCs, as compared to the untreated control (Figure 4A,A(i)). Although caspase-3 cleaved in MDA231 cells after treatment with the cells and secretome of DPMSCs, the total expression was not significantly different (*p* > 0.05) as compared to the untreated control (Figure 4B,B(i)). The phosphorylation of the Rb protein at serine 807 (S807) decreased significantly (*p* < 0.05) in MDA231 cells after treatment with DPMSCs’ cellular component or their secretome as compared to untreated control cells (Figure 4C,C(i)). Similarly, the MDA231 cell expression of Chk1 kinase after treatment with DPMSCs decreased significantly (*p* > 0.05) in the IC setting, while no significant change in expression levels was observed in SF and CM treated cells, as compared to the untreated control (Figure 4D,D(i)). Treatment of MDA321 cells with DPMSCs resulted in a significant increase (*p* > 0.05) in Chk2 in IC as compared to the SF, CM, and untreated controls ((Figure 4E,E(i)).

**Figure 5:**
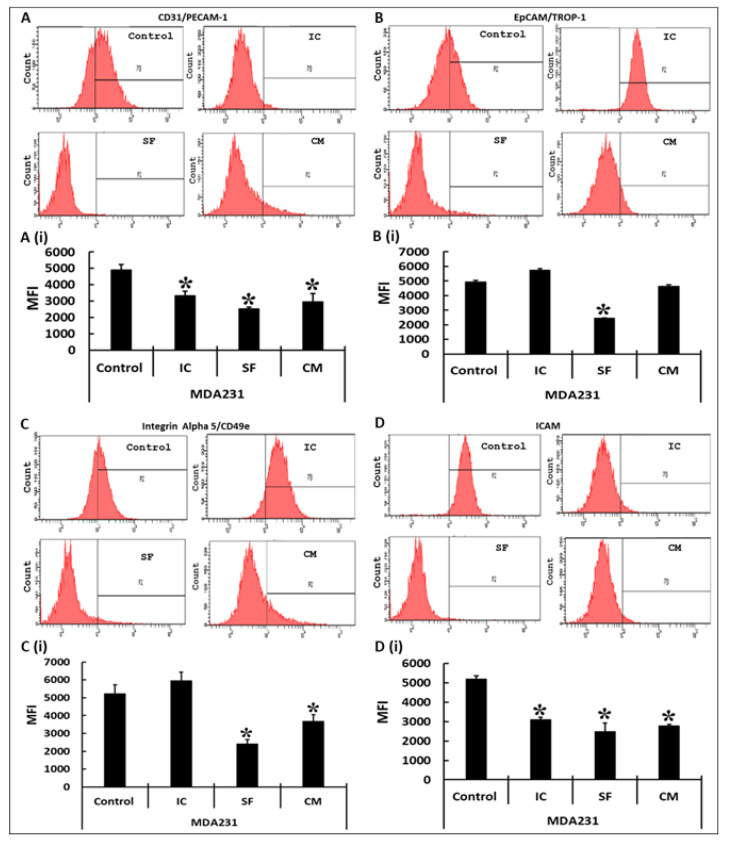
Effect of DPMSCs on MDA231 cell expression of adhesion molecules. Flow cytometry analysis of the adhesion molecules performed on DPMSC-treated MDA231 cells or untreated controls showed a significant decrease in the expression levels for PECAM in IC, SF, and CM settings as compared to the untreated control (**A**). EpCAM/TROP-1 was reduced significantly in MDA231 cells in the SF setting as compared to IC and CM settings, as well to the untreated control (**B**). The expression of integrin alpha 2/CD49e, showed a significant decrease in SF and CM treatment conditions (**C**), whereas ICAM1 was significantly reduced in all the treatment conditions tested (**D**). Bar graphs represent the data obtained from three independent experiments and are shown as mean florescence index (MFI) units pertaining to CD31/PECAM1 (**A**(**i**)), EpCAM/TROP1 (**B**(**i**)), integrin alpha 5/CD49e (**C**(**i**)), and ICAM1 (**D**(**i**)). Bars represent standard errors. * *p* < 0.05.

Various effectors responsible for the adhesion of MDA231 to its cellular matrix were assessed after their treatment with DPMSCs in IC and SF settings as well as after treatment with the CM of DPMSCs. In the flow cytometry assay the expression of CD31/PECAM1 in MDA231 cells decreased significantly after treatment with DPMSCs in both IC and SF settings of the cellular component as well as their secretome, as compared to the untreated control (Figure 5A,A(i)). The expression of EpCAM/TROP-1 in MDA231 cells treated with DPMSCs exhibited a significant reduction in the SF setting only, and interestingly it was slightly increased in the IC setting as compared to the control. Although MDA231 cells treated with 40% CM of DPMSCs showed a decrease in EpCAM/TROP-1 expression as compared to the untreated control, the decrease was not statistically significant (*p* > 0.05) (Figure 5B,B(i)). A similar trend was observed for integrin alpha 5/CD49e expression in DPMSC-treated MDA231 cells. As shown in Figure 5C,C(i), IC treatment did not alter the expression of integrin alpha 5/CD49e as compared to the untreated control. However, after treatment under SF conditions and with 40% CM of DPMSCs the MDA231 cells exhibited significantly reduced (*p* < 0.05) levels of integrin alpha 5/CD49e as compared to the untreated control (Figure 5C,C(i)). MDA231 cells treated with DPMSCs under IC, SF, and 40% CM of DPMSCs conditions showed a significant reduction in ICAM1 levels in all the treatment groups tested, as compared to the untreated control (Figure 5D,D(i)). The results of all these molecules were recorded as mean fluorescence index (MFI) units. 

## 4. Discussion

We have previously reported the isolation and characterization of human placental mesenchymal stem cells from the decidua region of the placenta, the decidua parietalis (DPMSCs). DPMSCs express and secrete several important factors, which have the ability to modify the functional activities of their target cells. They are multipotent cells that differentiate into multiple lineages and possess immunomodulatory properties [21]. DPMSCs induce natural killer cell expression of inflammatory receptors, and thus have the ability to enhance NK-cell-mediated anti-cancer activities [48]. We have recently reported that DPMSCs not only survive and function normally in the cancer microenvironment, but that their anti-cancer properties are enhanced in the harsh and toxic environment of a tumor [24]. These distinctive properties of DPMSCs make them an attractive therapy against inflammatory diseases such as cancer.

For their successful application as a therapy against cancer, we investigated the effects of the cellular component as well as the secretome of the DPMSCs on the functional consequences of the breast cancer cell line MDA231. In addition, the phenotypic and genotypic modulations were evaluated after the treatment of MDA231 cells with DPMSCs and the conditioned media obtained from them. The appropriate number of DPMSCs and the specific dose of the secretory component that has a measurable effect on the performance of cancer cells was necessary to be determined in a given time. These spatiotemporal effects were measured while treating the cancer cells directly with the DPMSCs or with the CM, for a specific period with a specific number of the cells and a specific dose of CM-DPMSCs, and measuring their effect. In order to differentiate between their effect on cancer and on normal tissues, our studies show that DPMSCs did not alter the survival of the HMECs, in IC, SF, or with the different concentrations of the CM. Interestingly, a co-culture of MDA231 cells with DPMSCs in IC and SF settings, as well as with treatment with 40% CM, resulted in a decrease in cellular viability, as observed in Figure 1. We have reported previously that, under the influence of cancer-conditioned media mimicking the cancer microenvironment, DPMSCs secrete a variety of anti-proliferative molecules, including IL-6, CCL13, CCL24, CXCL16, CSF1, and IL-1RN [24], which may have suppressed the cellular viability by reducing the proliferative potential of MDA231 cells when treated with DPMSCs in IC and SF settings, as observed in Figure 1A(i),A(ii). Moreover, normal HMECs did not create a conducive environment for DPMSCs to exert any such effect that may in turn be detrimental to them, and thus no change in cellular phenotype was observed, as shown in Figure 1B(i),B(ii). Interestingly, the treatment of MDA231 cells with the CM show a significant decrease (Figure 1A(iii)) in their overall viability as compared to the HMECs (Figure 1B(iii)) treated with the same doses under similar conditions. MSCs have been shown to secrete various factors, including cytokines, chemokines, growth factors, etc. Conditioned media obtained from such cells exert their influence on the target cells, resulting in immunomodulation, tissue regeneration, and anti-apoptosis [49]. However, the selective impact of CM-DPMSCs on the cancer cells and not on the normal primary cells may be because of the interaction of the unidentified components present in the CM, with the secretory factors of the cancer cells, that eventually bring about a change in their overall survival. However, further investigation is needed to identify the specific molecules involved in the modulation of MDA231 functions by the DPMSCs. 

A subtle decrease in viability in MDA231 cells after treatment with cellular or secretory products of the DPMSCs supports the concept that their sustained treatment may modulate other functional characteristics of the MDA231 cells. The suppression of adhesion molecules increases the detachment of cancer cells into the lymphatic or blood stream, which takes them into distant sites where they settle and proliferate, eventually resulting into a new tumor: the process called metastasis [50]. Adhesion between the cells and the extracellular matrix play a pivotal role in tissue architecture and integrity. It regulates cellular proliferation, cellular migration, and invasion, thereby regulating metastasis in various cancers [51]. A co-culture of DPMSCs with MDA231 cells in IC and SF settings, as well as their CM, did not alter the adhesion potential of the MDA231 cells significantly as compared to the untreated control (Figure 2A). As reported earlier [24], in the cancer microenvironment DPMSCs secrete pro-adhesive molecules, including BMP2, CX3CL1, and VEGFA, which may regulate the adhesive properties of MDA231 cells through their paracrine effect or through their secretome; this needs to be examined. Furthermore, FACS analysis exhibited a significant decrease in integrin alpha 5/CD49e, which is overexpressed in colon, breast, ovarian, lung, and brain tumors and is associated with a poor prognosis for patients [52]. The expression of the ICAM1 protein leads to the activation of multiple pathways with key roles in different stages of metastatic progression [53]. Soluble forms of ICAM1 enhance the pro-metastatic phenotype as well as pro-inflammatory and oncogenic signaling in lung and gastric cancer patients [54,55,56]. In addition, the modulation in expression of other adhesion molecules, including PECAM1 and EpCAM (Figure 5), suggest that DPMSCs stabilize the adhesion of MDA231 cells, thereby inhibiting the metastasis phenotypes. 

MDA231 cell proliferation reduced significantly after co-culturing with DPMSCs in IC and SF settings, as well as after treatment with their secretory products, as observed in Figure 2B. This is in accordance with the earlier reported results, where umbilical cord MSCs were shown to reduce proliferation and induce apoptosis in the U251 human glioma cell line [57]. In addition, it has been reported that a co-culture of MSCs isolated from various sources suppresses tumor growth and proliferation in brain [18,57,58,59,60], breast [61,62,63], lung [57], colorectal [57,64], ovarian [65], and esophageal [63] cancers. MSCs in therapy are considered as a double-edged sword, with both immunosuppressive as well as immune-enhancing properties that depend upon the tissue of their origin as well as the target tissue or pathological condition [66]. Although MSCs isolated from many tissues have shown favorable responses against cancer and other diseases, as described above, the outcome may not be favorable with every MSC type used in every disease setting. One such example is that bone marrow MSCs have been shown to contribute to the adaptation and invasiveness of breast cancer cells in skeletal tissues [67]. These findings suggest that utmost care must be taken while selecting MSCs for therapeutic purposes, as they may prove counterproductive in certain settings. 

The upregulation of various tumor suppressor genes and downregulation of various oncogenes were observed in MDA231 cells after their co-culturing with DPMSCs, as well as after treatment with their secretory products (Table 1). Among them include important tumor suppressor genes, such as BRCA1 and BRCA2 [31], FOXA1 and GATA3 [32], NME1 [33], PTEN [34], RARB [35], CDKN1A, CDKN2A [36], TP53 [38], etc. CDKN1A, also called as P21, binds to and inhibits the activity of cyclin-dependent kinase (CDK) complexes. Its expression is controlled by the tumor suppressor protein p53, and it mediates the p53-dependent cell cycle G1 phase arrest [68]. CDKN2A or ARF stabilizes the tumor suppressor protein p53 and sequesters E3 ubiquitin-protein ligase (MDM2), which is responsible for the degradation of p53 [69]. A decrease in functional characteristics, including proliferation in MDA231 cells treated with DPMSCs, may be mediated through the p53-CDKN1A-CDKN2A pathways. However, further studies need to be performed to validate it. Among the most prominent downregulated genes in MDA231 cells after their treatment with DPMSCs are MUC1 [41], XBP1 [42], CTSD [43], ABCB1 [44], etc. Modulation in the expression of these genes may play an important role in regulating the proliferation of MDA231 cells co-cultured with DPMSCs. The exact mechanism underlying their pro-proliferative effects on DPMSCs will be examined in a future study.

The peculiar feature of advanced stage cancer is its metastasis to neighboring or distant tissues and organs. The dissemination of cancer cells from the primary site to other organs as well as their survival and proliferation increase the chances of mortality in cancer patients [70]. In metastasis, the cancer cells utilize their cellular migration and invasion properties to detach from the primary site, their intravasation into the blood and lymphatic system, and invade distant tissues and develop into a secondary tumor [71,72]. Increased migration and invasive properties require the expression of important genes responsible for cell motility [73]. A co-culture of MDA231 cells in an IC or SF setting as well as their treatment with the secretome of DPMSCs resulted in a significant decrease in their migration as well as invasive phenotypes (Figure 2C,D and Figure 3A,B). This in in accordance with our previous report that cancer-conditioned media modulate the expression of numerous genes in DPMSCs, which are involved in cellular migration and invasion. It has been reported that human cord blood MSCs decrease the cellular invasion and migration of a glioblastoma cell line through their downregulation of PI3K/AKT, c-Myc/ERK, and EGFR/c-Met activities [59]. Involvement of Wnt signaling has also been reported to hamper the cellular migration of breast cancer by MSCs isolated from adipose and human umbilical cord tissues [74,75].

Angiogenesis or neovascularization is another important step during oncogenesis, and is an important hallmark in cancer development and progression [76]. Since DPMSCs did not undergo angiogenesis as they failed the tube formation assay, we assessed if they have an impact on the angiogenic properties of MDA231 cells. Co-culturing (IC) as well as the secretory factors in the CM significantly inhibited the tube formation potential of MDA231 cells (Figure 3C). Our results are in agreement with similar studies performed on glioblastoma cells using human bone marrow as well as human cord blood MSCs. Both the bone marrow as well the cord blood MSCs not only inhibited tumor growth, but also decreased angiogenesis in glioblastoma cell lines involving IL1β, FAK, and AKT pathways [77,78].

MDA231 cells, after treatment with DPMSCs, showed a significant decrease in proliferation in IC and SF settings, as well as after treatment with the CM of DBMSCs. In order to dissect the mechanism behind this phenotype, we evaluated the expression status of a few cell effector molecules involved in cell cycle progression and apoptosis pathways. mRNA profiling of the MDA231 cells after treatment with DPMSCs exhibited modulation in the expression of multiple effector molecules with pro-apoptotic and cell checkpoint roles (data not shown). Proteomic analysis of targets such as p53, caspase-3, phosphor-Rb (S807), and Chk1 as well as Chk2 was performed in MDA231 cells after treatment with DBMSCs, for validation purposes. The results demonstrated a significant increase in the expression of p53, repression in the phosphorylation of pRB, a significant decrease in Chk1 expression, and a significant increase in Chk2 expression. In addition, the treated cells demonstrated cleavage of caspase-3, a strong indicator of an apoptosis pathway (Figure 4). However, it is not clear if any one specific or all the pathways mediate the pro-apoptotic signals in DPMSC-treated MDA231 cells. p53 is a tumor suppressor gene that functions to inhibit the proliferation of abnormal cells, thus suppressing neoplastic development [38]. It activates various anti-proliferative pathways by activating or restraining the functions of key effector genes involved [79]. Hypo-phosphorylated RB binds and sequesters the E2F/DP family of transcription factors, restricting the transcription of genes responsible for the G1-to-S phase transition of the cells. This cell cycle inhibitory function is abrogated when pRB undergoes phosphorylation at S807 [80,81]. Chk1 phosphorylates the main regulators during DNA damage, rendering them inactivated, thus blocking G2/M transition. It is also responsible for DNA damage response, S and M phases, and G2/M transition cell phases in the cell cycle [82]. Chk2, a serine/threonine kinase, is a key component of the DNA damage response. It is activated under various cellular stresses and induces the cellular response, including cell cycle checkpoint activation, the induction of apoptosis, or senescence [83]. Similarly, caspase-3 plays a key role in cellular proliferation by activating calcium-independent phospholipase A2, which increases the synthesis and release of PGE_2_ through arachidonic acid. PGE_2_ has been reported to increase tumor growth in many cancers, including colon, prostate, breast, lung, etc. [84].

The overall results of this study suggest that DPMSCs, as well as their secretome, induces the loss of functional capabilities, including apoptosis in the MDA231 cell line, in vitro. The results indicate that DPMSCs may be considered as a major cornerstone in the field of cellular therapy, in parallel to CAR T-cell and similar therapies. 

The limitation of this study is that it is performed in a lab setting and the results obtained are purely based on the in vitro studies. The authenticity of these findings must be validated in preclinical studies in animals, followed by phase I clinical trials. Therefore, these studies may need to be executed in animal models to further validate their efficiency, anti-tumor mechanism of action, safety profiles, pharmacokinetics, and pharmacodynamics before their application in human cancer therapies.

## 5. Conclusions

In this study, we investigated the usability and feasibility of the DPMSCs and their secretory products as therapeutic agents against cancer. Both the cellular component and the conditioned media isolated from the DPMSCs did not alter the adhesion of MDA231 cells, but inhibited the proliferation of MDA231 cells. In addition, DPMSCs restricted other functional activities of MDA231 cells, including migration, invasion, and angiogenesis. Under the influence of DPMSCs and their secretome, the MDA231 cells expressed upregulation of multiple pro-apoptotic genes and downregulation of many oncogenes, explaining the mechanism behind their anti-tumor properties. These data demonstrate the possibility of using DPMSCs as therapeutic agents against cancer. However, a more comprehensive study plan including preclinical studies are needed to further confirm their therapeutic potential and to assess their pharmacokinetics as well as pharmacodynamics for cancer treatment purposes.

## Figures and Tables

**Table 1:** The modulation of gene expression in MDA231 cells after treatment with DPMSCs.

**Tumor Suppressor Genes**						
**Gene Symbol**	**Gene Name**	**Function**	**Expression**			**References**
			**IC**	**SF**	**CM**	
BRCA1	Breast cancer gene 1	Tumor suppressor	Up	Up	Up	[31]
BRCA2	Breast cancer gene 2	Tumor suppressor	Up	Up	Up	[31]
FOXA1	Forkhead box protein A1	Tumor suppressor	Up	Up	Up	[32]
GATA3	GATA binding protein 3	Tumor suppressor	Up	Up	Up	[32]
NME1	NME/NM23 nucleoside diphosphate kinase 1	Tumor suppressor	Up	Up	Up	[33]
PTEN	Phosphatase and tensin homolog	Tumor suppressor	Up	Up	Up	[34]
RARB	Retinoic acid receptor b	Tumor suppressor	Up	Up	Up	[35]
CDKN1A	Cyclin-dependent kinase inhibitor 1A	Tumor suppressor	Up	Up	NSC	[36]
CDKN2A	Cyclin-dependent kinase inhibitor 2A	Tumor suppressor	Up	NSC	Up	[36]
CDKN1C	Cyclin-dependent kinase inhibitor 1C	Tumor suppressor	NSC	Up	Up	[36]
CDH13	Cadherin 13, H-cadherin	Tumor suppressor	Up	NSC	Up	[37]
TP53	Tumor protein p53	Tumor suppressor	Up	NSC	Up	[38]
ESR2	Estrogen receptor beta	Tumor suppressor	NSC	Up	NSC	[39]
APC	Adenomatous polyposis coli	Tumor suppressor	NSC	NSC	Up	[40]
	**Oncogenes**					
**Gene Symbol**	**Gene Name**	**Function**	**Expression**			**References**
			**IC**	**SF**	**CM**	
MUC1	Mucin 1	Oncogene	Down	NSC	Down	[41]
XBP1	X-box binding protein 1	Oncogene	Down	NSC	NSC	[42]
CTSD	Cathepsin-D	Oncogene	NSC	Down	Down	[43]
ABCB1	ATP-binding cassette sub-family B member 1	Oncogene	NSC	Down	NSC	[44]
CCNA1	Cyclin A1	Oncogene	NSC	NSC	Down	[45]
ID1	Inhibitor of differentiation/DNA binding 1	Oncogene	NSC	NSC	Down	[46]
SERPINE1	Plasminogen activator inhibitor-1	Oncogene	NSC	NSC	Down	[47]

Up: upregulation; Down: downregulation; and NSC: no significant change observed.

## Data Availability

All data and materials will be available upon request.

## References

[1] Sung H., Ferlay J., Siegel R.L., Laversanne M., Soerjomataram I., Jemal A., Bray F. (2021). Global Cancer Statistics 2020: GLOBOCAN Estimates of Incidence and Mortality Worldwide for 36 Cancers in 185 Countries. CA. Cancer J. Clin..

[2] Goffin J., Lacchetti C., Ellis P.M., Ung Y.C., Evans W.K. (2010). First-line systemic chemotherapy in the treatment of advanced non-small cell lung cancer: A systematic review. J. Thorac. Oncol. Off. Publ. Int. Assoc. Study Lung Cancer.

[3] Chen Z., Fillmore C.M., Hammerman P.S., Kim C.F., Wong K.-K. (2014). Non-small-cell lung cancers: A heterogeneous set of diseases. Nat. Rev. Cancer.

[4] Sawyers C. (2004). Targeted cancer therapy. Nature.

[5] Sermer D., Brentjens R. (2019). CAR T-cell therapy: Full speed ahead. Hematol. Oncol..

[6] Gomes J.P.A., Assoni A.F., Pelatti M., Coatti G., Okamoto O.K., Zatz M. (2017). Deepening a Simple Question: Can MSCs Be Used to Treat Cancer?. Anticancer Res..

[7] Javan M.R., Khosrojerdi A., Moazzeni S.M. (2019). New Insights into Implementation of Mesenchymal Stem Cells in Cancer Therapy: Prospects for Anti-angiogenesis Treatment. Front. Oncol..

[8] Nwabo Kamdje A.H., Kamga P.T., Simo R.T., Vecchio L., Seke Etet P.F., Muller J.M., Bassi G., Lukong E., Goel R.K., Amvene J.M. (2017). Mesenchymal stromal cells’ role in tumor microenvironment: Involvement of signaling pathways. Cancer Biol. Med..

[9] Phinney D.G., Sensebé L. (2013). Mesenchymal stromal cells: Misconceptions and evolving concepts. Cytotherapy.

[10] Oswald J., Boxberger S., Jørgensen B., Feldmann S., Ehninger G., Bornhäuser M., Werner C. (2004). Mesenchymal stem cells can be differentiated into endothelial cells in vitro. Stem Cells.

[11] Atiya H., Frisbie L., Pressimone C., Coffman L. (2020). Mesenchymal Stem Cells in the Tumor Microenvironment. Adv. Exp. Med. Biol..

[12] Qiao L., Xu Z., Zhao T., Zhao Z., Shi M., Zhao R.C., Ye L., Zhang X. (2008). Suppression of tumorigenesis by human mesenchymal stem cells in a hepatoma model. Cell Res..

[13] Nakamura K., Ito Y., Kawano Y., Kurozumi K., Kobune M., Tsuda H., Bizen A., Honmou O., Niitsu Y., Hamada H. (2004). Antitumor effect of genetically engineered mesenchymal stem cells in a rat glioma model. Gene Ther..

[14] Maestroni G.J., Hertens E., Galli P. (1999). Factor(s) from nonmacrophage bone marrow stromal cells inhibit Lewis lung carcinoma and B16 melanoma growth in mice. Cell. Mol. Life Sci..

[15] Qiao C., Xu W., Zhu W., Hu J., Qian H., Yin Q., Jiang R., Yan Y., Mao F., Yang H. (2008). Human mesenchymal stem cells isolated from the umbilical cord. Cell Biol. Int..

[16] Otsu K., Das S., Houser S.D., Quadri S.K., Bhattacharya S., Bhattacharya J. (2009). Concentration-dependent inhibition of angiogenesis by mesenchymal stem cells. Blood.

[17] Gondi C.S., Veeravalli K.K., Gorantla B., Dinh D.H., Fassett D., Klopfenstein J.D., Gujrati M., Rao J.S. (2010). Human umbilical cord blood stem cells show PDGF-D-dependent glioma cell tropism in vitro and in vivo. Neuro-Oncol..

[18] Akimoto K., Kimura K., Nagano M., Takano S., To’a Salazar G., Yamashita T., Ohneda O. (2013). Umbilical cord blood-derived mesenchymal stem cells inhibit, but adipose tissue-derived mesenchymal stem cells promote, glioblastoma multiforme proliferation. Stem Cells Dev..

[19] Basmaeil Y., Al Subayyil A., Abumaree M., Khatlani T. (2021). Conditions Mimicking the Cancer Microenvironment Modulate the Functional Outcome of Human Chorionic Villus Mesenchymal Stem/Stromal Cells in vitro. Front. Cell Dev. Biol..

[20] Abomaray F.M., Al Jumah M.A., Alsaad K.O., Jawdat D., Al Khaldi A., Alaskar A.S., Al Harthy S., Al Subayyil A.M., Khatlani T., Alawad A.O. (2016). Phenotypic and Functional Characterization of Mesenchymal Stem/Multipotent Stromal Cells from Decidua Basalis of Human Term Placenta. Stem Cells Int..

[21] Abumaree M.H., Abomaray F.M., Alshehri N.A., Almutairi A., AlAskar A.S., Kalionis B., Al Jumah M.A. (2016). Phenotypic and Functional Characterization of Mesenchymal Stem/Multipotent Stromal Cells from Decidua Parietalis of Human Term Placenta. Reprod. Sci..

[22] Abumaree M.H., Al Jumah M.A., Kalionis B., Jawdat D., Al Khaldi A., AlTalabani A.A., Knawy B.A. (2013). Phenotypic and functional characterization of mesenchymal stem cells from chorionic villi of human term placenta. Stem Cell Rev. Rep..

[23] Abumaree M.H., Abomaray F.M., Alshabibi M.A., AlAskar A.S., Kalionis B. (2017). Immunomodulatory properties of human placental mesenchymal stem/stromal cells. Placenta.

[24] Bahattab E., Khatlani T., Abomaray F.M., Messaoudi S.A., Abumaree M.H. (2019). Cancer Conditioned Medium Modulates Functional and Phenotypic Properties of Human Decidua Parietalis Mesenchymal Stem/Stromal Cells. Tissue Eng. Regen. Med..

[25] Al Subayyil A., Basmaeil Y.S., Alenzi R., Khatlani T. (2021). Human Placental Mesenchymal Stem/Stromal cells (pMSCs) inhibit agonist-induced platelet functions reducing atherosclerosis and thrombosis phenotypes. J. Cell. Mol. Med..

[26] Ke N., Wang X., Xu X., Abassi Y.A. (2011). The xCELLigence system for real-time and label-free monitoring of cell viability. Methods Mol. Biol..

[27] Vistejnova L., Dvorakova J., Hasova M., Muthny T., Velebny V., Soucek K., Kubala L. (2009). The comparison of impedance-based method of cell proliferation monitoring with commonly used metabolic-based techniques. Neuro Endocrinol. Lett..

[28] Stefanowicz-Hajduk J., Ochocka J.R. (2020). Real-time cell analysis system in cytotoxicity applications: Usefulness and comparison with tetrazolium salt assays. Toxicol. Rep..

[29] Dowling C.M., Herranz Ors C., Kiely P.A. (2014). Using real-time impedance-based assays to monitor the effects of fibroblast-derived media on the adhesion, proliferation, migration and invasion of colon cancer cells. Biosci. Rep..

[30] Basmaeil Y., Al Rashid M., Khatlani T., AlShabibi M., Bahattab E., Abdullah M.L., Abomaray F., Kalionis B., Massoudi S., Abumaree M. (2020). Preconditioning of Human Decidua Basalis Mesenchymal Stem/Stromal Cells with Glucose Increased Their Engraftment and Anti-diabetic Properties. Tissue Eng. Regen. Med..

[31] Zheng L., Li S., Boyer T.G., Lee W.H. (2000). Lessons learned from BRCA1 and BRCA2. Oncogene.

[32] Albergaria A., Paredes J., Sousa B., Milanezi F., Carneiro V., Bastos J., Costa S., Vieira D., Lopes N., Lam E.W. (2009). Expression of FOXA1 and GATA-3 in breast cancer: The prognostic significance in hormone receptor-negative tumours. Breast Cancer Res..

[33] Pamidimukkala N., Puts G.S., Kathryn Leonard M., Snyder D., Dabernat S., De Fabo E.C., Noonan F.P., Slominski A., Merlino G., Kaetzel D.M. (2021). Nme1 and Nme2 genes exert metastasis-suppressor activities in a genetically engineered mouse model of UV-induced melanoma. Br. J. Cancer.

[34] Csolle M.P., Ooms L.M., Papa A., Mitchell C.A. (2020). PTEN and Other PtdIns(3,4,5)P(3) Lipid Phosphatases in Breast Cancer. Int. J. Mol. Sci..

[35] Doan T.B., Cheung V., Clyne C.D., Hilton H.N., Eriksson N., Young M.J., Funder J.W., Muscat G.E.O., Fuller P.J., Clarke C.L. (2020). A tumour suppressive relationship between mineralocorticoid and retinoic acid receptors activates a transcriptional program consistent with a reverse Warburg effect in breast cancer. Breast Cancer Res..

[36] DiPippo A.J., Patel N.K., Barnett C.M. (2016). Cyclin-Dependent Kinase Inhibitors for the Treatment of Breast Cancer: Past, Present, and Future. Pharmacotherapy.

[37] Andreeva A.V., Kutuzov M.A. (2010). Cadherin 13 in cancer. Genes. Chromosom. Cancer.

[38] Gasco M., Shami S., Crook T. (2002). The p53 pathway in breast cancer. Breast Cancer Res..

[39] Omoto Y., Iwase H. (2015). Clinical significance of estrogen receptor β in breast and prostate cancer from biological aspects. Cancer Sci..

[40] Furuuchi K., Tada M., Yamada H., Kataoka A., Furuuchi N., Hamada J.I., Takahashi M., Todo S., Moriuchi T. (2000). Somatic mutations of the APC gene in primary breast cancers. Am. J. Pathol..

[41] Kufe D.W. (2013). MUC1-C oncoprotein as a target in breast cancer: Activation of signaling pathways and therapeutic approaches. Oncogene.

[42] Gomez B.P., Riggins R.B., Shajahan A.N., Klimach U., Wang A., Crawford A.C., Zhu Y., Zwart A., Wang M., Clarke R. (2007). Human X-box binding protein-1 confers both estrogen independence and antiestrogen resistance in breast cancer cell lines. FASEB J. Off. Publ. Fed. Am. Soc. Exp. Biol..

[43] Garcia M., Platet N., Liaudet E., Laurent V., Derocq D., Brouillet J.P., Rochefort H. (1996). Biological and clinical significance of cathepsin D in breast cancer metastasis. Stem Cells.

[44] Leonessa F., Clarke R. (2003). ATP binding cassette transporters and drug resistance in breast cancer. Endocr. Relat. Cancer.

[45] Syed Khaja A.S., Dizeyi N., Kopparapu P.K., Anagnostaki L., Härkönen P., Persson J.L. (2013). Cyclin A1 Modulates the Expression of Vascular Endothelial Growth Factor and Promotes Hormone-Dependent Growth and Angiogenesis of Breast Cancer. PLoS ONE.

[46] Gumireddy K., Li A., Kossenkov A.V., Cai K.Q., Liu Q., Yan J., Xu H., Showe L., Zhang L., Huang Q. (2014). ID1 promotes breast cancer metastasis by S100A9 regulation. Mol. Cancer Res..

[47] Yang J.-D., Ma L., Zhu Z. (2019). SERPINE1 as a cancer-promoting gene in gastric adenocarcinoma: Facilitates tumour cell proliferation, migration, and invasion by regulating EMT. J. Chemother..

[48] Abumaree M.H., Bahattab E., Alsadoun A., Al Dosaimani A., Abomaray F.M., Khatlani T., Kalionis B., El-Muzaini M.F., Alawad A.O., Alaskar A.S. (2018). Characterization of the interaction between human decidua parietalis mesenchymal stem/stromal cells and natural killer cells. Stem Cell Res. Ther..

[49] Zhuang W.Z., Lin Y.H., Su L.J., Wu M.S., Jeng H.Y., Chang H.C., Huang Y.H., Ling T.Y. (2021). Mesenchymal stem/stromal cell-based therapy: Mechanism, systemic safety and biodistribution for precision clinical applications. J. Biomed. Sci..

[50] Moh M.C., Shen S. (2009). The roles of cell adhesion molecules in tumor suppression and cell migration: A new paradox. Cell Adh. Migr..

[51] Gumbiner B.M. (1996). Cell Adhesion: The Molecular Basis of Tissue Architecture and Morphogenesis. Cell.

[52] Schaffner F., Ray A.M., Dontenwill M. (2013). Integrin α5β1, the Fibronectin Receptor, as a Pertinent Therapeutic Target in Solid Tumors. Cancers.

[53] Benedicto A., Romayor I., Arteta B. (2017). Role of liver ICAM-1 in metastasis. Oncol. Lett..

[54] Sprenger A., Schardt C., Rotsch M., Zehrer M., Wolf M., Havemann K., Heymanns J. (1997). Soluble intercellular adhesion molecule-1 in patients with lung cancer and benign lung diseases. J. Cancer Res. Clin. Oncol..

[55] Maruo Y., Gochi A., Kaihara A., Shimamura H., Yamada T., Tanaka N., Orita K. (2002). ICAM-1 expression and the soluble ICAM-1 level for evaluating the metastatic potential of gastric cancer. Int. J. Cancer.

[56] Witkowska A.M., Borawska M.H. (2004). Soluble intercellular adhesion molecule-1 (sICAM-1): An overview. Eur. Cytokine Netw..

[57] Yang C., Lei D., Ouyang W., Ren J., Li H., Hu J., Huang S. (2014). Conditioned media from human adipose tissue-derived mesenchymal stem cells and umbilical cord-derived mesenchymal stem cells efficiently induced the apoptosis and differentiation in human glioma cell lines in vitro. BioMed Res. Int..

[58] Jiao H., Guan F., Yang B., Li J., Shan H., Song L., Hu X., Du Y. (2011). Human umbilical cord blood-derived mesenchymal stem cells inhibit C6 glioma via downregulation of cyclin D1. Neurol. India.

[59] Velpula K.K., Dasari V.R., Tsung A.J., Gondi C.S., Klopfenstein J.D., Mohanam S., Rao J.S. (2011). Regulation of glioblastoma progression by cord blood stem cells is mediated by downregulation of cyclin D1. PLoS ONE.

[60] Kološa K., Motaln H., Herold-Mende C., Koršič M., Lah T.T. (2015). Paracrine effects of mesenchymal stem cells induce senescence and differentiation of glioblastoma stem-like cells. Cell Transplant..

[61] Ganta C., Chiyo D., Ayuzawa R., Rachakatla R., Pyle M., Andrews G., Weiss M., Tamura M., Troyer D. (2009). Rat umbilical cord stem cells completely abolish rat mammary carcinomas with no evidence of metastasis or recurrence 100 days post-tumor cell inoculation. Cancer Res..

[62] Gauthaman K., Yee F.C., Cheyyatraivendran S., Biswas A., Choolani M., Bongso A. (2012). Human umbilical cord Wharton’s jelly stem cell (hWJSC) extracts inhibit cancer cell growth in vitro. J. Cell. Biochem..

[63] Dzobo K., Vogelsang M., Thomford N.E., Dandara C., Kallmeyer K., Pepper M.S., Parker M.I. (2016). Wharton’s Jelly-Derived Mesenchymal Stromal Cells and Fibroblast-Derived Extracellular Matrix Synergistically Activate Apoptosis in a p21-Dependent Mechanism in WHCO1 and MDA MB 231 Cancer Cells In Vitro. Stem Cells Int..

[64] Yuan Y., Zhou C., Chen X., Tao C., Cheng H., Lu X. (2018). Suppression of tumor cell proliferation and migration by human umbilical cord mesenchymal stem cells: A possible role for apoptosis and Wnt signaling. Oncol. Lett..

[65] Khalil C., Moussa M., Azar A., Tawk J., Habbouche J., Salameh R., Ibrahim A., Alaaeddine N. (2019). Anti-proliferative effects of mesenchymal stem cells (MSCs) derived from multiple sources on ovarian cancer cell lines: An in-vitro experimental study. J. Ovarian Res..

[66] Li W., Ren G., Huang Y., Su J., Han Y., Li J., Chen X., Cao K., Chen Q., Shou P. (2012). Mesenchymal stem cells: A double-edged sword in regulating immune responses. Cell Death Differ..

[67] Najar M., Fayyad-Kazan H., Faour W.H., Badran B., Journe F., Lagneaux L. (2017). Breast cancer cells and bone marrow mesenchymal stromal cells: A regulated modulation of the breast tumor in the context of immune response. Inflamm. Res..

[68] Wagner M., Klussmann J.P., Fangmann R., Linder R., Elewa M.E., Eidt S., Rose V.M., Jungehulsing M., Schulze H.J. (2001). Cyclin-dependent kinase-inhibitor 1 (CDKN1A) in the squamous epithelium of the oropharynx: Possible implications of molecular biology and compartmentation. Anticancer Res..

[69] Seo J., Seong D., Lee S.R., Oh D.-B., Song J. (2020). Post-Translational Regulation of ARF: Perspective in Cancer. Biomolecules.

[70] Chambers A.F., Groom A.C., MacDonald I.C. (2002). Dissemination and growth of cancer cells in metastatic sites. Nat. Rev. Cancer.

[71] Yamaguchi H., Wyckoff J., Condeelis J. (2005). Cell migration in tumors. Curr. Opin. Cell Biol..

[72] Friedl P., Wolf K. (2003). Tumour-cell invasion and migration: Diversity and escape mechanisms. Nat. Rev. Cancer.

[73] Wang W., Goswami S., Lapidus K., Wells A.L., Wyckoff J.B., Sahai E., Singer R.H., Segall J.E., Condeelis J.S. (2004). Identification and testing of a gene expression signature of invasive carcinoma cells within primary mammary tumors. Cancer Res..

[74] Liu J., Han G., Liu H., Qin C. (2013). Suppression of cholangiocarcinoma cell growth by human umbilical cord mesenchymal stem cells: A possible role of Wnt and Akt signaling. PLoS ONE.

[75] Visweswaran M., Arfuso F., Dilley R.J., Newsholme P., Dharmarajan A. (2018). The inhibitory influence of adipose tissue-derived mesenchymal stem cell environment and Wnt antagonism on breast tumour cell lines. Int. J. Biochem. Cell Biol..

[76] Furuya M., Yonemitsu Y. (2008). Cancer neovascularization and proinflammatory microenvironments. Curr. Cancer Drug Targets.

[77] Dasari V.R., Kaur K., Velpula K.K., Dinh D.H., Tsung A.J., Mohanam S., Rao J.S. (2010). Downregulation of Focal Adhesion Kinase (FAK) by cord blood stem cells inhibits angiogenesis in glioblastoma. Aging (Albany NY).

[78] Ho I.A.W., Toh H.C., Ng W.H., Teo Y.L., Guo C.M., Hui K.M., Lam P.Y.P. (2013). Human bone marrow-derived mesenchymal stem cells suppress human glioma growth through inhibition of angiogenesis. Stem Cells.

[79] Zilfou J.T., Lowe S.W. (2009). Tumor suppressive functions of p53. Cold Spring Harb. Perspect. Biol..

[80] Tamrakar S., Rubin E., Ludlow J.W. (2000). Role of pRB dephosphorylation in cell cycle regulation. Front. Biosci..

[81] Vélez-Cruz R., Johnson D.G. (2017). The Retinoblastoma (RB) Tumor Suppressor: Pushing Back against Genome Instability on Multiple Fronts. Int. J. Mol. Sci..

[82] Patil M., Pabla N., Dong Z. (2013). Checkpoint kinase 1 in DNA damage response and cell cycle regulation. Cell. Mol. Life Sci..

[83] Zannini L., Delia D., Buscemi G. (2014). CHK2 kinase in the DNA damage response and beyond. J. Mol. Cell Biol..

[84] Donato A.L., Huang Q., Liu X., Li F., Zimmerman M.A., Li C.-Y. (2014). Caspase 3 promotes surviving melanoma tumor cell growth after cytotoxic therapy. J. Investig. Dermatol..

